# Aortic valve stenosis after previous coronary bypass: Transcatheter valve implantation or aortic valve replacement?

**DOI:** 10.1186/1749-8090-7-47

**Published:** 2012-05-29

**Authors:** Olivier Jegaden, Joel Lapeze, Fadi Farhart, Guy de Gevigney

**Affiliations:** 1 Department of Cardiac Surgery and Transplantation, Hospital Louis Pradel, University Claude Bernard Lyon 1, INSERM Carmen, 28 Avenue du doyen LEPINE, Bron, 69677, France

## Abstract

We report a prospective comparison between transcatheter valve implantation (TAVI, n = 13) and surgical aortic valve replacement (AVR, n = 10) in patients with severe aortic valve stenosis and previous coronary bypass surgery (CABG). All patients had at least bilateral patent internal thoracic arteries bypass without indication of repeat revascularization. After a similar post-procedure outcome, despite one early death in TAVI group, the 1-year survival was 100% in surgical group and in transfemoral TAVI group, and 73% in transapical TAVI group. When previous CABG is the lone surgical risk factor, indications for a TAVI procedure have to be cautious, specially if transfemoral approach is not possible.

## Background

Aortic Valve surgery (AVR) after previous coronary artery bypass (CABG) is always challenging and usually the indication of redo surgery is delayed because of the risk of reoperation in old patients with patent arterial grafts. Few years ago, we have described a surgical approach through an inferior T hemisternotomy for aortic valve surgery in patients with an in situ right internal mammary artery to left anterior descending artery passing in front of the ascending aorta, allowing a good surgical exposure and providing good results with an adapted surgical strategy [1]. Since the introduction of transcatheter aortic valve implantation (TAVI), these patients are now referred to this alternative therapeutic option as high risk patients with an adverse thorax even if they only present technical challenges to conventional AVR [2]. We report a prospective comparison between TAVI and AVR using our technique in patients with severe aortic valve stenosis (AS) and previous CABG.

## Methods

From May 2009 and December 2010, 23 patients with AS and previous CABG were referred to our department for a TAVI procedure. Mean age was 76 ± 9 years (55–88), mean logistic Euroscore was 25 ± 15 (5.8–52) and mean delay after CABG was 11 ± 5 years (0.2–19). All patients had at least both IMA grafting and all arterial grafts were patent without indication for repeat revascularization. After the screening, 13 patients underwent a TAVI procedure with the implantation of a Sapien prothesis (Edwards life-sciences, Irvine, CA) using a transapical approach (TA) in 9 or a transfemoral approach (TF) in 4, according to the available vascular access, and 10 patients underwent a AVR procedure with the implantation of a Magna bioprothesis (Edwards life-sciences, Irvine, CA): 3 patients had refused the “new” TAVI procedure and 7 patients had a too large aortic annulus (>25 mm). In both group (TAVI and AVR), patients were similar in age (76 ± 11 vs 76 ± 6,) and logistic Euroscore (25 ± 14 vs 25 ± 16 respectively). In TAVI group, the impairment of left ventricular ejection fraction was higher (49 ± 12 vs 57 ± 9, ns) and the delay from CABG surgery was shorter (9 ± 6 vs 14 ± 2, *p* < 0.01); in this group, 3 patients had a severe porcelain aorta and in 2 of them, AS was known at the time of CABG surgery and the decision of a further TAVI procedure was decided during the off-pump CABG procedure in front of a “untouchable” aorta. Written informed consent was obtained from the patient for publication of this report and any accompanying images.

## Results

All TAVI procedures were done in catheterization laboratory under general anesthesia. In one patient with porcelain aorta, an intra-ventricular migration of the prothesis occurred during the TA procedure, leading to the implantation of a second valve and then a surgical removal of the first prothesis was successfully done through a right mini-thoracotomy using a left atrial and trans-mitral approach under beating heart. All other TAVI and AVR procedures were successfully done. One patient after TAVI procedure died on day 20^th^ from general weakness; he was 88-year old with logistic euroscore 52 and the indication was probably overtaken. The outcomes of the patients are summarized in Table 1. After AVR, extubation time was significantly longer and Tropinin level (24^th^ hours) was significantly higher. Transfusion requirement was higher after AVR (ns). After TAVI, pacemaker implantation was higher (ns) and 2 patients had a paravalvular leak (grade 2). ICU stay and hospital stay were similar in both group. There was no major adverse event in both group as myocardial infraction, stroke or vascular complication. The mean follow-up was 1.2 year; one sudden death occurred 3 months after a TAVI procedure. At 1 year, actuarial survival was 100% in AVR group and 84 ± 21% in TAVI group (ns): 100% in TF and 78 ± 28% in TA (ns).

**Table 1:** Outcome of patients after the procedure

	**AVR n = 10**	**TAVI n = 13**	**p**
Extubation time (hours)	6.5 ± 2.8	2.8 ± 1.9	<0,02
ICU Stay (Days)	1.2 ± 0.8	1 ± 0.8	ns
Tropinin (24th hours)	6.7 ± 7	3 ± 1.2	ns
Tranfusion requirement (%)	70%	38%	ns
Pacemaker implantation (%)	20%	54%	ns
Paravalvular leak (%)	0%	15%	ns
30-day Mortality (%)	0%	7.5%	ns
Hospitalstay (days)	11 ± 2,5	10 ± 3	ns
MACCE (Infarctus, stroke)	0 (%)	0 (%)	ns

AVR, Aortic valve replacement; TAVI, transcatheter aortic valve implantation; ICU, Intensive care unit; MACCE, Major adverse cardiac and cerebrovascular event

## Comments

Patients with AS after previous CABG are often distinguished by high risk factors for AVR: elderly, symptomatic heart failure, long history of ischemic cardiomyopathy with left ventricular impairment. Redo-surgery is technically challenging regarding the surgical approach, the myocardial protection, the calcified aortic root, and specially in case of patent arterial grafts. However, conventional AVR as a redo procedure after CABG with patent grafts can be performed with excellent results and a lower mortality than estimated [3], even in case of both IMA grafts, thanks to the use of an adapted surgical strategy [1]. TAVI procedure with its less invasive nature has been believed to offer a safer treatment solution for high risk patients [2] and we could expect to observe a benefit impact of TAVI in the specific situation of patients with previous CABG. According to our short series, the advantage of TAVI in comparison with AVR is not obvious. Early mortality and post-procedure outcome are quite similar: earlier extubation time, lower Troponin level and higher rate of pacemaker implantation after TAVI have to be balanced against higher rate of transfusion and no paravalvular leak after AVR. The 1-year survival are the same after AVR and transfemoral TAVI (100%) and lower after transapical TAVI (78%) as if TA approach was more aggressive than AVR for the ischemic and impaired underlying myocardium. Concomitant coronary artery disease has been demonstrated as a significant risk factor for mortality in patients having TAVI [4]; in our series, the choice of operative approach, either TA or TF, was found to be a risk factor of mortality. In the randomized PARTNER trial [5], TAVI and AVR were associated with similar rates of survival at 1-year; however, the results of the subgroup analyses suggested that TAVI was associated with higher mortality than AVR among patients with a history of CABG regardless the operative approach for TAVI; by experience, we could imagine that TA procedure had been more frequent in this subgroup.

In an observational study, Drews et al. [6] reported that previous heart surgery was not a risk factor in TA-TAVI: early mortality and 1-year survival were similar in patients with or without previous surgery, but the 1.5 year survival observed was 73% in TAVI as first procedure and 52% in TAVI as second procedure. Recently, Ducrocq et al. [7] evaluated the impact of previous CABG on the outcome of patients after TAVI procedure; conversely, previous CABG was identified as an independent predictive factor of better mid-trem survival, which is maybe related to residual bias in an observational study; however their study confirmed that TA approach is more frequent in previous CABG group (43% vs 26%) and that TA approach is a predictor of 2-year mortality.

Paradoxically, these data are not conflicting with the results of the randomized PARTNER trial [5] and with the short contribution of our series: 1) In comparison with vascular approach, TA approach is a risk factor in TAVI procedure, 2) The impact of previous CABG on the outcome of patients after TAVI remains controversial, but seems to be, 3) In patients with previous CABG and eligible for TAVI or AVR, surgical replacement is maybe better than TA-TAVI.

## Limitations

Our series presents several limitations, mainly the lack of randomization and the small number of patients. However, it emphasizes the hypothesis from the PARTNER trial [5] that surgical replacement is maybe better than TA-TAVI in patients with previous CABG.

## Conclusion

Our series confirms that if TAVI can be considered as a good alternative to AVR in patients with previous CABG in order to avoid technical challenges of conventional surgery, it is maybe not the best option when previous CABG is the lone surgical risk factor for AVR and when transfemoral TAVI is not possible. Nowadays, in such patients without a porcelain aorta, we are thinking about a transaortic approach for TAVI procedure, through a right anterior mini thoracotomy (2^nd^ ICS), as an alternative to the transapical approach. Another alternative could be the subclavian approach but it is not suitable to Sapien prothesis.
